# How to split a G-quadruplex for DNA detection: new insight into the formation of DNA split G-quadruplex†
†Electronic supplementary information (ESI) available: DNA sequence, Fig. S1–S11, Tables S1–S4, extended experimental details and discussions. See DOI: 10.1039/c5sc01287b



**DOI:** 10.1039/c5sc01287b

**Published:** 2015-06-02

**Authors:** Jinbo Zhu, Libing Zhang, Shaojun Dong, Erkang Wang

**Affiliations:** a State Key Laboratory of Electroanalytical Chemistry , Changchun Institute of Applied Chemistry , Chinese Academy of Sciences , Changchun 130022 , P. R. China . Email: ekwang@ciac.ac.cn; b University of Chinese Academy of Sciences , Beijing , 100049 , P. R. China

## Abstract

Here, we get a new insight into the formation of a split G-quadruplex from the viewpoints of the split mode and guanine base number. An unusual result is that the split mode 4 : 8 performed best in six split modes, including the frequently used mode 1 : 3 and 2 : 2 in the split G-quadruplex enhanced fluorescence assay. Circular dichroism spectra verified the conclusion. The application of the split G-quadruplex based assay in DNA detection was performed on the point mutations of the JAK2 V617F and HBB genes. A multi-target analysis method based on a pool of G-segments split from T30695 (GGGTGGGTGGGTGGGT) by the magic “law of 4 : 8” was established.

## Abstract 2

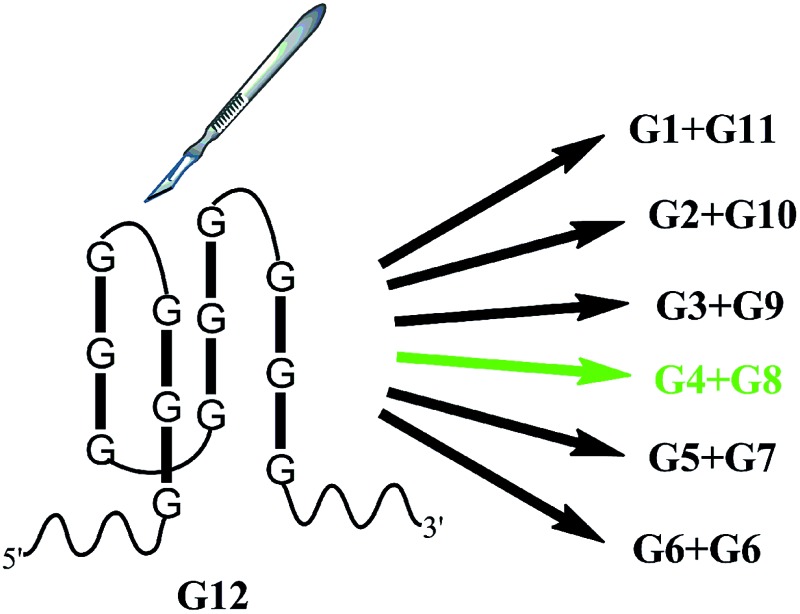
A magic “law of 4 : 8” to split the G-quadruplex for DNA detection has been found.

## Introduction

The G-quadruplex is an alternative DNA motif with a special four-stranded structure, that has shown great application potential in molecular biology, biomedicine, analytical chemistry and DNA computing.^1–7^ In this structure, four guanine bases associate *via* Hoogsteen hydrogen-bonding to form the so-called guanine tetrad, and then two or more guanine tetrads stack on top of each other to form a G-quadruplex.^8,9^ Since the G-quadruplex can dramatically enhance the catalytic ability of hemin (known as G-quadruplex DNAzyme) and the fluorescence of some porphyrin derivatives [*e.g.*, protoporphyrin IX (PPIX), mesoporphyrin IX (MPIX), *N*-methyl mesoporphyrin IX (NMM)], it has been widely used as a signal amplifier in various biosensors.^10–17^ Recently, as a recombination G-quadruplex structure, split G-quadruplex has been introduced and applied as a binary probe in many fields for its flexible structure and design.^18–26^ In this strategy, the guanine bases of the G-quadruplex are often distributed on two different strands for the target strand to drive them together by hybridization to reproduce the G-quadruplex and induce an increase in the catalytic ability or fluorescence.

However, in the reported papers the separation is often done in the loop part of the G-quadruplex, thus, the twelve guanine bases of G-quadruplex, such as PW17 or T30965, are always divided into two halves by the ratio of either 2 : 2 or 1 : 3 (*i.e.* 6 : 6 or 3 : 9 for the 12 guanine bases).^23,24,27–31^ In addition, the number of guanine bases of the whole split G-quadruplex is often twelve. What will happen if the separation is done between the guanine bases and more or less guanine bases are contained in the split G-quadruplex? In other words, we are curious about the conditions where the G-quadruplex is divided in other ratios, such as 4 : 8, 2 : 10, 1 : 11, *etc.*, and 11 or 13 guanine bases are used to form the split G-quadruplex. A better way to split the G-quadruplex may be found from them. Meanwhile, a split G-quadruplex based DNA sensor with a lower background and higher fluorescent signal may be obtained this way, which would be very significant for DNA sensing and single nucleotide polymorphism (SNP) detection. Moreover, we can acquire further information about the interaction between the G short segments and unveil the factors that affect the formation of the DNA split G-quadruplex. Stimulated by the desire to uncover these questions, we carry out our research about how to effectively split a G-quadruplex for biosensing.

## Results and discussion

### Comparison of different G-quadruplex split modes

**Scheme 1:**
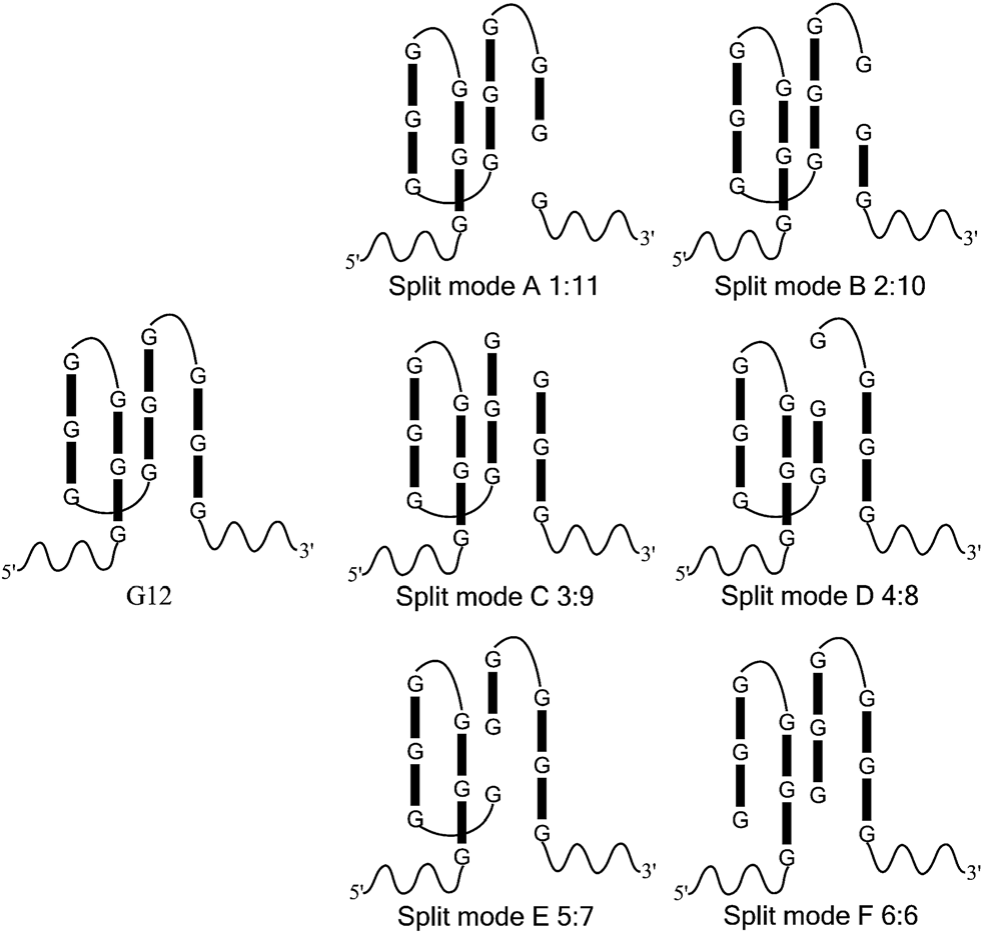
Splitting the G-quadruplex by six different modes. It should be noted that the schematic figure only indicates the split site of T30695, and it does not represent the real DNA structure in solution.

**Fig. 1:**
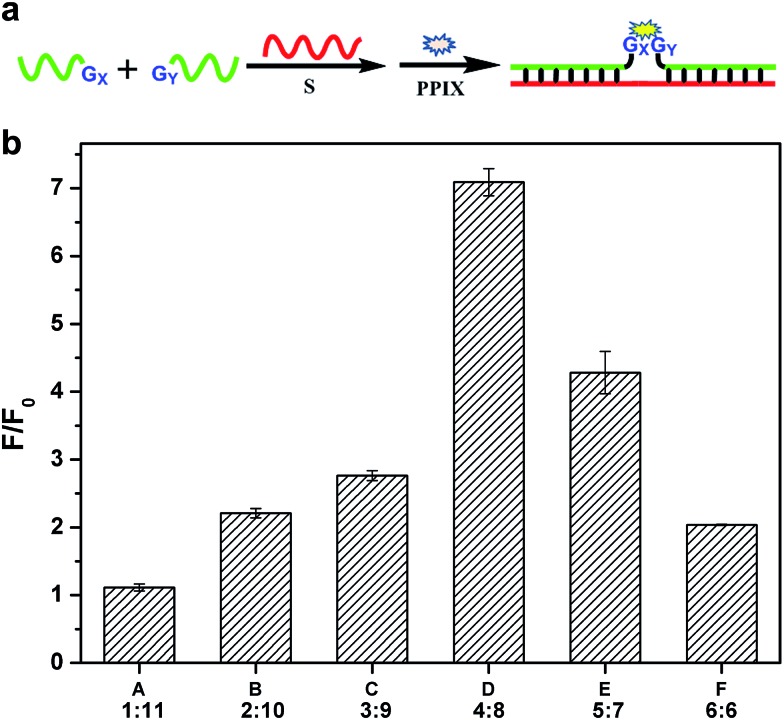
(a) Schematic diagram of the split G-quadruplex enhanced fluorescence assay for DNA detection. S represents the target strand. G*x* and G*y* represent the G segments that are linked to the binding region. *x* and *y* represent the number of the guanine base. (b) Ratio of signal to background for the six different split modes in the split G-quadruplex enhanced fluorescence assay. The data were gained from three independent experiments.

In this work, we took a typical G-quadruplex sequence T30695 (GGGTGGGTGGGTGGGT) as an example to compare the different split modes. All six possible ways to split a G-quadruplex composed of twelve guanine bases are illustrated in Scheme 1 (in fact, taking the orientation of the DNA strand into consideration, there will be twelve different ways to split the G-quadruplex, in total. This point will be discussed later). These six split modes are named as split mode A (1 : 11), B (2 : 10), C (3 : 9), D (4 : 8), E (5 : 7) and F (6 : 6), respectively. As a binary probe, each G-rich segment is linked with an analyte binding arm. As shown in Fig. 1a, the target strand will hybridize with them and drive the guanine bases together to form the split G-quadruplex. A G-quadruplex binding molecule PPIX was chosen to report the formation of the G-quadruplex in this work. PPIX usually aggregates into micelles with low fluorescence in aqueous solution, whereas its fluorescence can be dramatically enhanced after binding to a G-quadruplex.^32–34^ Thus, for this split G-quadruplex enhanced fluorescence assay, a high fluorescence signal will be produced when the target strand is present to induce the formation of the split G-quadruplex. However, the fluorescence will remain at a low level when the target sequence is absent, or SNP occurs on the target DNA.

**Fig. 2:**
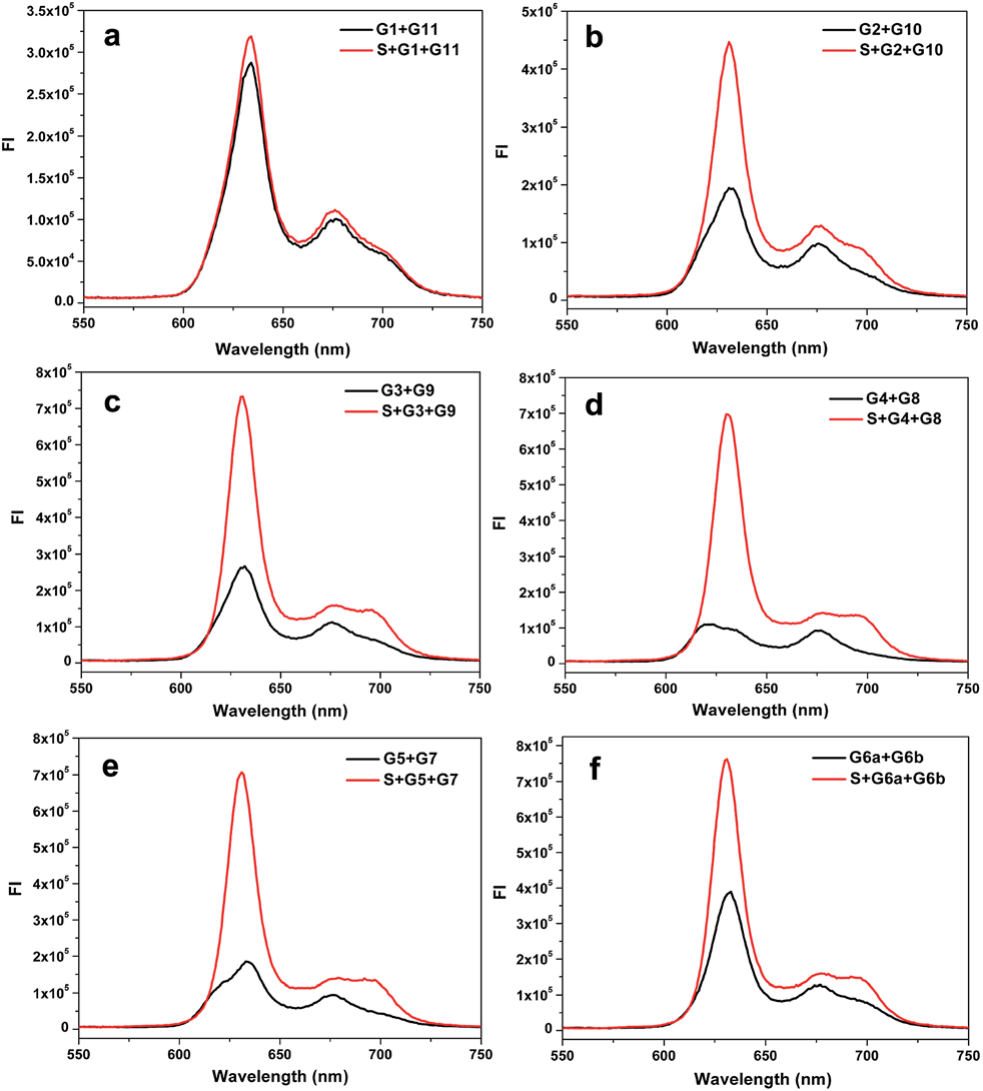
Fluorescence emission spectra of the complexes of PPIX and DNA G-segments split by the different modes. The six plots show the backgrounds and signals of the six split modes: (a) split mode A, (b) split mode B, (c) split mode C, (d) split mode D, (e) split mode E, (f) split mode F. The strands used for each curve have been indicated in the figure.

In fact, the native fluorescence of PPIX is very weak in solution. A slight increase in the fluorescence will appear after the addition of the G-rich segments. This fluorescence intensity (FI) can be treated as the background and the other one enhanced by the split G-quadruplex can be regarded as the target signal. The ratio of the signal to background varies considerably for the different split modes. Obviously, the larger the ratio gained in detection is, the better. To figure out the optimal way to split the G-quadruplex, we tested all six split modes (Fig. 2). The signal to background ratios of the different modes are provided in Fig. 1b. Surprisingly, the performances of the two frequently employed modes (C and F) are both moderate. Instead, the largest ratio is obtained from the mode D (4 : 8). Some reasons could be identified to explain this phenomenon by analyzing the FI data of each mode. We can classify these six modes into three types, according to the FI data. Mode A and B belong to type 1, in which the target signal was quite low (Fig. 2a and b). In mode A, there is only one guanine base on strand G1, which might be too short to draw the attention of eleven guanine bases on G11 to form the complete G-quadruplex. Thus, the structure of the split G-quadruplex is still imperfect and the fluorescence signal is weak in this case. For mode B, although the condition is better than mode A, the strength of the signal is still poor, compared with the other ones. The small number of guanine bases on G2 could be the reason for this result. It is worth noting that the absence of only one guanine base (strand G11) would intensively hinder the binding between PPIX and the G-quadruplex. Mode F (6 : 6) should belong to type 2, in which the background is very high (Fig. 2f). Even though the signal is high enough to indicate the presence of the target strand, the high background greatly affects the sensitivity of this assay. We deduced that the two G-rich segments, generated by cleaving the G-quadruplex 50 : 50, are easy to bind together by themselves and hence the FI is relatively high without strand S.^35–37^ The remaining three modes can be categorized into a third type. In this group, the backgrounds are all at a low level and the high signals are easy to obtain (Fig. 2c–e), thus these modes are more suitable for use as probes to detect the target sequence. Mode D is the best of them, because its background is the lowest. The new issues are why is the 4 : 8 split mode the best and why is its background signal the lowest?

### Background signals of G-segments

**Fig. 3:**
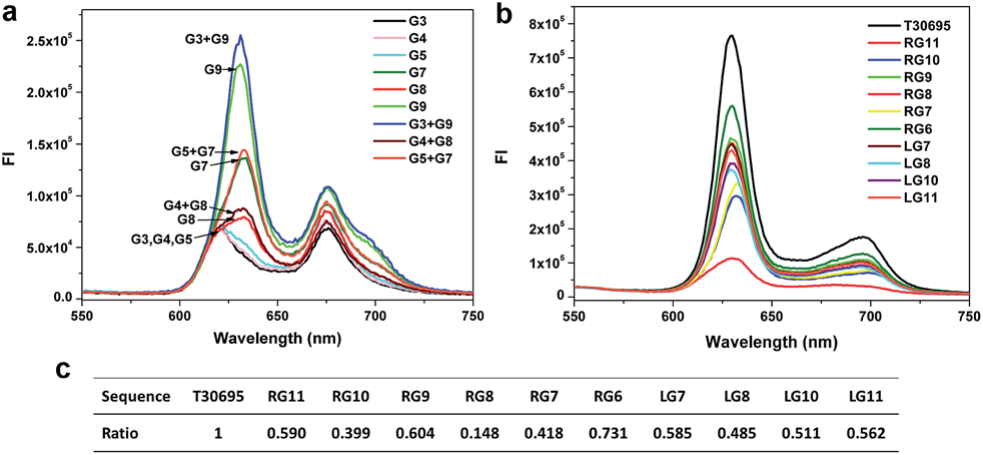
(a) and (b) Fluorescence emission spectra of the complexes of PPIX and different DNA G-segments. The strands used for each curve have been indicated in the plots. (c) Relative fluorescence intensity ratio of the FI of the G segment to that of T30695 at 630 nm.

As we know, when the target strand is absent, the background fluorescent signal is mainly induced by the interaction between the PPIX and the self-assembled G-quadruplex or single G-rich strand. To further confirm the source of the background signal in each split mode, we investigated the FIs induced by different G-segments. As shown in Fig. 3a, the FIs aroused by the short G-rich strands G3, G4 and G5 are very weak, whereas the FIs of the long G-rich strands G7, G8 and G9 are obviously higher than the previous strands and close to those of their respective mixed groups. The results demonstrate that the main sources of the backgrounds for the corresponding split modes are caused by the long G-rich strand, rather than the interaction between the short and long G-rich strands. The FI induced by G8 is the lowest in the three long G-rich strands. Split mode D should benefit greatly from this point, and so it has the lowest background compared with the other modes. A comparison of the FIs caused by T30695 and long G-rich strands and their native polyacrylamide gel electrophoresis (PAGE) analysis are given in section S2 of the ESI.†

To avoid the effects of the random sequence linked to the G-rich bases, we investigated the FIs of PPIX induced by the pure long G segments without sensing arms. The long G segments generated from splitting the T30695 sequence from the 5′ end were also tested here. Similar results were gained in Fig. 3b and c. What is interesting at first is that the magic “law of 4 : 8” for the low background signal also works well in this case. The signal of the strand RG8 that contains eight guanine bases is still the lowest, which is very different with the high signal of strand RG6. The high background FI of split mode F is due to the self-assembled dimeric G-quadruplex composed by the six-guanine-base contained strand, which is easy to bind together to form the G-quadruplex with the help of a certain amount of potassium ions.^25,38^ From this viewpoint, the low signal of strand G8 may be partly due to the weak tendency of the eight-guanine-base contained strand to bind together with itself to form a dimeric G-quadruplex. The affinity of PPIX with the three G segments RG7, RG8 and RG9 are shown in Fig. S3.† It is indeed harder for the eight-guanine-base contained strand RG8 to bind with PPIX compared with the other G-segments. Additionally, when the long G segments are gained by splitting T30695 from the 5′ end, the signal of LG8 (generated from 4 : 8 split mode) is still the lowest compared with LG7, RG9, LG10 and LG11 in Fig. 3c. However, splitting the G-quadruplex from the 3′ end is apparently better, because the RG8 outputs such a low background signal. Furthermore, application of the 4 : 8 mode on other G-quadruplex and effects of DNA concentrations, salt ions and number of guanine bases in split G-quadruplex were all investigated and are given in section S3–5 of ESI.† Our research demonstrated that the background signals of the eight-guanine-base contained G segments split from other G-quadruplexes (PW17 and 306T2) were still the lowest compared with the other modes, potassium ion played a key role in the formation of the split G-quadruplex for split mode D and E, and the four-stranded structure can form only when the number of guanine bases is equal to or greater than 12.

### Circular dichroism spectroscopy and melting studies

**Fig. 4:**
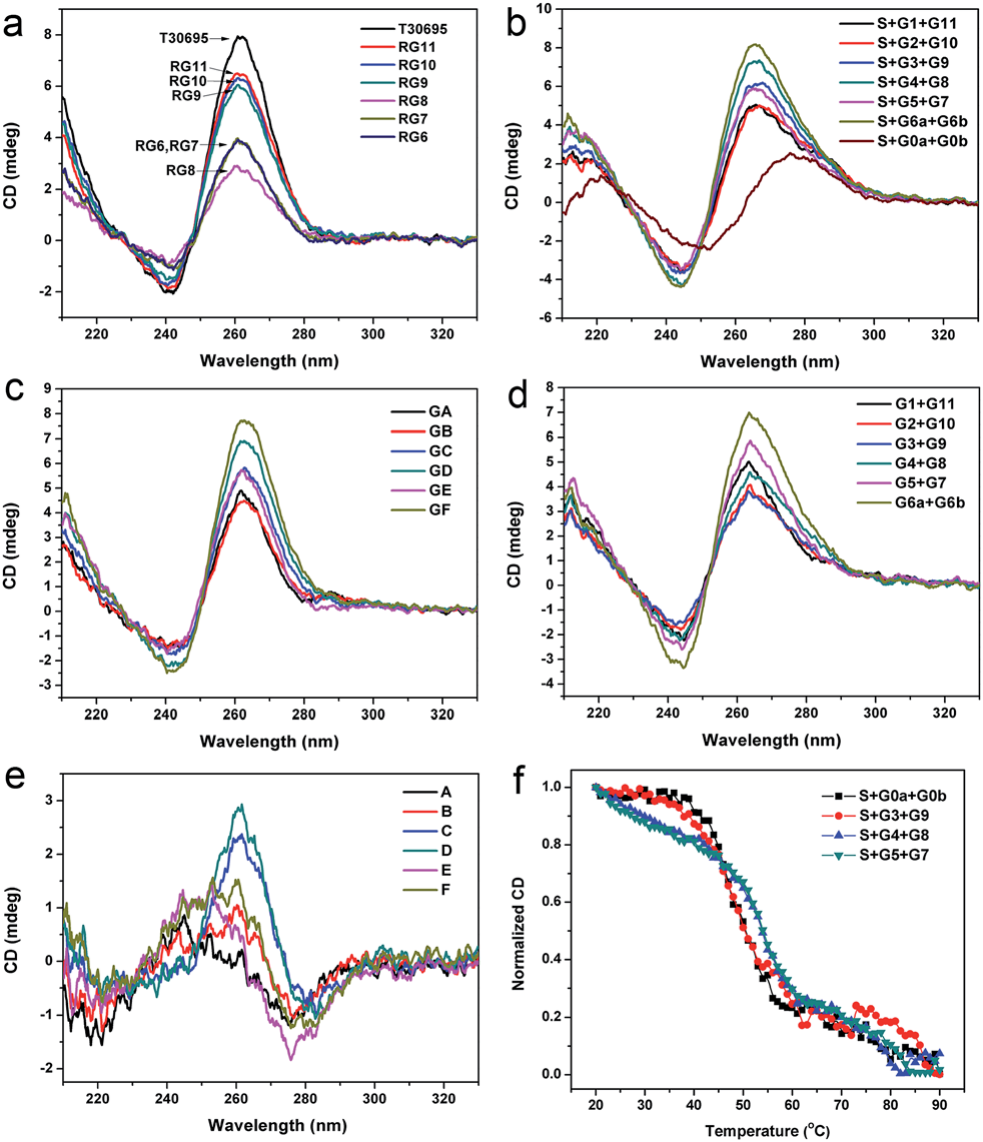
CD spectra of the G segments and six different split modes. Strands added in each sample in (a), (b) and (d) have been indicated in the figure. The relative reference CD values for different split modes in (c) and (e) have also been indicated in the figure. (f) CD melting curves for four different DNA complexes (S + G*x* + G*y*) at 276 nm in lithium cacodylate buffer. Concentrations of the DNA strands were 10 μM in (a), 5.0 μM in (b) and (d) and 2.0 μM in (f).

Since a G-quadruplex owns unique circular dichroism (CD) characteristics, CD can also be used to identify the formation of the G-quadruplex structure.^24,39,40^ CD spectra of the G-rich strands without the binding region are shown in Fig. 4a. For T30965, there is a characteristic positive peak around 260 nm in its’ CD spectra. The signals at 260 nm of these G segments are all lower than the integrated G-quadruplex strand, which indicates that the missing guanine bases seriously influence the formation of the G-quadruplex structure. Strand RG8 shows the lowest signal at the characteristic positive peak in these strands, which should be ascribed to its low formation rate of the G-quadruplex structure. The dimeric G-quadruplex in the solution of RG6 makes a significant contribution to its CD signal at 260 nm and helps it surpass the signal of strand RG8. Additionally, we investigated the spectra of the three-strand complexes; these results are shown in Fig. 4b. Since a double helix and parallel G-quadruplex possess similar typical peaks in the CD spectra, two segments without a guanine base (G0a and G0b) were used to bind with S to show the bands of the duplex helix.^19,24^ By subtracting the data of S + G0a + G0b from the other curves, we obtained the spectra of the split G-quadruplex for the different split modes (Fig. 4c). The positions of the positive and negative peaks match the reported characteristic peaks of the parallel G-quadruplex, which proves that the four-stranded structures are well formed in these complexes. The peaks of modes D and F are obviously higher than the others in Fig. 4c, but the background of the self-assembled G segments cannot be ignored (Fig. 4d). The high CD signal of the G segments of mode F corresponds to its high fluorescence background in Fig. 2f, which supports our presumption that the symmetrically split G-segments are easier to self-assemble into a G-quadruplex than the other split modes. By subtracting the CD signal of the G-segments from the corresponding curves in Fig. 4c, we produced the relative reference value in Fig. 4e. Since the background part has been removed, this reference value can truly reflect the sensitivity of each mode and there is a good agreement with the fluorescence results that the performance of the split mode D is the best in the CD experiment.

The formation of the split G-quadruplex is mainly dependent on the hybridization of the three-strand complex (S + G*x* + G*y*). In turn, the binary G-quadruplex structure will also increase the stability of the whole DNA complex. To identify this assumption and compare the stability of the different split G-quadruplexes based on the different split modes, we obtained the CD thermal denaturation profiles of four different DNA complexes. The main part of the whole DNA complex is the double helix structure, whose CD characteristic positive peak is at 276 nm, so we firstly monitored the change in the CD signal at this wavelength. As shown in Fig. 4f, the DNA complexes based on split modes D and E are more stable than the complex without the G-rich sequence (S + G0a + G0b), which proves that the binary G-quadruplex structure also enhances the stability of the whole DNA complex in return. The UV and CD melting curves of these DNA complexes at 265.5 nm were also collected to study the change of the G-quadruplex structure and similar results were obtained (see section S6 of the ESI†). The melting temperatures (*T*_m_) evaluated from the CD and UV melting curves are given in Table S2.†

### Application in DNA detection

**Fig. 5:**
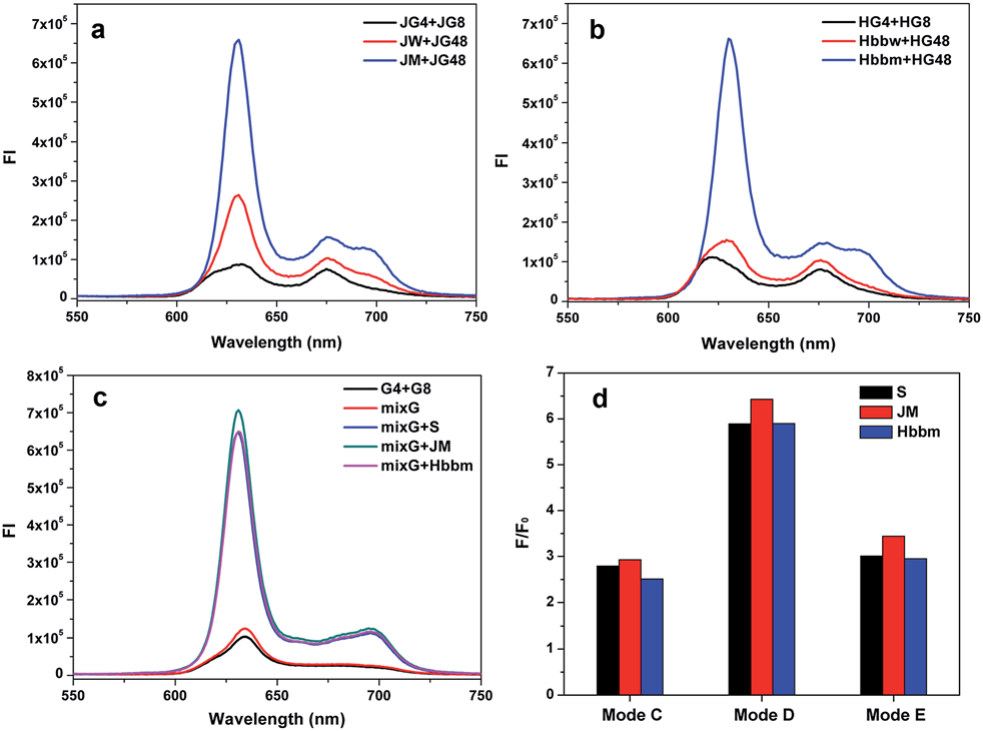
Fluorescence emission spectra of the complexes of PPIX and DNA G-segments split by mode D for the detection of JAK2 V617F (a) and HBB (b) point mutation. The strands added for each curve have been indicated in the plots. Hbbm and Hbbw refer to mutation and wild type of the HBB gene segments, respectively. (c) Analysis of multiple target strands by a pool of G-segments. MixG represents a group of G-segments split by mode D, which consists of strands G4, G8, JG4, JG8, HG4 and HG8. (d) Ratio of signal to background for three different split modes in the detection of multiple target strands by a pool of G-segments.

To investigate the practical application of the different split modes in the detection of DNA and SNP, a Janus kinase 2 (JAK2) V617F mutation was chosen as a model. Chronic myeloproliferative disorders (MPDs) have been identified to be closely associated with an acquired mutation in the JAK2 gene.^41,42^ The point mutation (JAK2 V617F; nucleotide G > T) causes a valine to phenylalanine substitution, resulting in constitutive activation of the JAK2 protein and overproduction of abnormal blood cells. The analysis of this mutation has been endorsed by the World Health Organization (WHO) for diagnosing these disorders. In this work, the wild and mutation sequence segments of the JAK2 gene were named JW and JM, respectively. We planned to detect this mutation using the split mode D (4 : 8) at first. The G-segments were designed based on mode D to bind JM and emit a high alarm fluorescence signal to report the mutation (JG4 and JG8, Table S1†). However, for the wild type sequence (strand JW), the signal would stay at a low level. The data are shown in Fig. 5a. The mutation strand can also induce high fluorescence signals in other split modes (modes C and E, Fig. S10†). The difference is that the background of the G-segments split by mode D is lower than those of the others and the signal to background ratio of mode D is the highest (Fig. S10 and Table S3†). This phenomenon proves that split mode D is still the optimal strategy in the six modes, even though the sequences of the binding domain are changed. Thus, our conclusion about the split modes is held true on a wider scale. For SNP detection, the signal differences between JM and JW are similar for these three modes (Table S3†). Thus, the split styles has little affect on SNP detection and the three modes can all be used to detect the JAK2 V617F mutation. The well-known point mutation in the β-globin (HBB) gene that would cause sickle-cell anemia was also detected by the corresponding G-rich probes designed on the basis of the split mode D (Fig. 5b).^43,44^ This result demonstrates the wide application potential of the optimal split mode D (4 : 8) based split G-quadruplex enhanced fluorescence assay in DNA and SNP detection.

Furthermore, benefiting from the low background of the G-segments generated based on the split mode D (4 : 8), we could put an arsenal of G-segments against different targets together, to build a multiple target strands sensor. Here, we chose the three different target strands S, JM and Hbbm as model targets and collected their G-segments probes, split by 4 : 8 together for sensing. As shown in Fig. 5c, the presence of any target strand would give rise to the high fluorescence signal. Since the background of the G-segments split by the mode D is low enough, there is no big change on the background after the congregation of the G-segments for the different targets. For the other split modes, the fluorescent backgrounds are higher and the differences aroused by the targets are less obvious than that of mode D (Fig. 5d and S11†). Thus, a pool of various G-segments split by the mode D would be an outstanding multi-target analysis tool. Moreover, it also provides an ideal solution to construct a multi-input OR logic gate with a low background, as we have done in our previous work.^21^

## Conclusions

In this work, we gained a new insight into the formation of the split G-quadruplex from the perspectives of the split mode and guanine base number. We have inspected the influences of the different split modes on the split G-quadruplex enhanced PPIX fluorescence assay and found that the split mode 4 : 8 has the highest signal to background ratio. The low fluorescence emission of PPIX in the presence of eight-guanine-base contained G-rich strand leads to the success of the magic “law of 4 : 8” for splitting a G-quadruplex. Thanks to its low background, this mode may be a super strategy for DNA detection or working as a signal readout in some logic devices, compared with the other split modes such as 1 : 3 or 2 : 2 frequently used before.^18,21,45,46^ The CD results support our conclusion from another perspective. We further investigated the effects of DNA concentration, salt ions and guanine base number for the split G-quadruplex enhanced fluorescence assay. The point mutation strands of the JAK2 V617F and HBB genes have both been analyzed by the split G-quadruplex enhanced fluorescence assay. The performances of the modes C, D and E in SNP detection did not vary hugely, but the background of mode D was the lowest, even though the sequences of the hybridized parts had changed. We could put these G-segments split by the mode D for different target strands together, to build a multi-target analysis method. Overall, the G-quadruplex split by this optimal split mode (4 : 8) can be applied in a wide area and the new insight for the formation of the split G-quadruplex will provide useful guidance for future studies.

## Experimental section

### Materials

The DNA strands were purchased from the Sangon Biotechnology Co., Ltd (Shanghai, China) and their sequences and functions are listed in Tables S1† and 1, respectively. PPIX was purchased from Sigma-Aldrich. Other chemicals were of reagent grade and were used without further purification. The DNA strands were dissolved in water as a stock solution and were quantified by UV-vis absorption spectroscopy on a Cary 60 UV-vis Spectrophotometer (Varian, USA).

**Table 1:** Table of abbreviations used for different DNA strands and their functions in experiments

Abbreviations	Functions
S	A target strand that can drive G segments together through hybridization with sensing arms in G segments
G*x*/G*y* ^*a*^	G segments split from T30695 with a sensing arm for detection of strand S
RG*x* ^*a*^	Long G segments gained by splitting T30695 from the 3′ end
LG*x* ^*a*^	Long G segments gained by splitting T30695 from the 5′ end
S + G*x* + G*y* ^*a*^	Three-strand complex formed upon the hybridization of sensing arms
JW/JM	Wild (JW) and mutation (JM) type target segments of JAK2 gene
JG*x* ^*a*^	G segments split from T30695 with a sensing arm for detection of JM
Hbbw/Hbbm	Wild (Hbbw) and mutation (Hbbm) type target segments of HBB gene
HG*x* ^*a*^	G segments split from T30695 with a sensing arm for detection of Hbbm
mixG^*a*^	A mixture solution of G*x*, G*y*, HG*x*, HG*y*, JG*x* and JG*y* for detection of S, Hbbm and JM at the same time

^*a*^“*x*” or “*y*” represents the number of guanine bases in corresponding G segment. The value of them depends on the split mode.

### Fluorescence spectroscopic analysis

The oligonucleotides stock solutions were diluted with Tris buffer (5 mM Tris–HCl, 0.5 mM EDTA, 100 mM NaCl, 20 mM KCl, 10 mM MgCl_2_, pH 8.0) for hybridization. The DNA solutions were heated at 88 °C for 10 min and slowly cooled down to room temperature (15 °C). Then, a freshly prepared PPIX solution with Tris buffer was added to the DNA solution and the mixture was incubated for 1 h before a fluorescence test. The fluorescent analysis was performed in the Tris buffer with a final concentration of 0.5 μM for PPIX, 0.3 μM for strands G*x*, G*y* and S (G*x* and G*y* represent the G-rich strands that hybridize with S, like G3, G9, *etc.*). The detection of the point mutations in the JAK2 V617F and HBB genes was performed in a similar way. A Fluoromax-4 spectrofluorometer (HORIBA Jobin Yvon, Inc., NJ) was used to record the fluorescence emission spectra of the DNA–PPIX complexes from 550 to 750 nm, with an excitation wavelength of 410 nm.
