# Supplementary material for: How to split a G-quadruplex for DNA detection: new insight into the formation of DNA split G-quadruplex
Source: Chem Sci. 2015 Jun 2;6(8):4822–7. doi: 10.1039/c5sc01287b (PMC5667574; doi:10.1039/c5sc01287b)
Supplement: Supplementary file 1 [file SC-006-C5SC01287B-s001.pdf]

Electronic Supplementary Material (ESI) for Chemical Science.
This journal is © The Royal Society of Chemistry 2015

Electronic Supplementary Information

How to split a G-quadruplex for DNA detection: new insight into the formation of DNA split G-quadruplex

Jinbo Zhu,[ab] Libing Zhang,[a] Shaojun Dong[a] and Erkang Wang*[a]

[a] *State Key Laboratory of Electroanalytical Chemistry, Changchun Institute of Applied Chemistry, Chinese Academy of Sciences, Changchun 130022, P. R. China.*

[b] *University of Chinese Academy of Sciences, Beijing, 100049, P. R. China.*

*To whom correspondence should be addressed. Tel: +86-431-85262003. E-mail: ekwang@ciac.ac.cn

S1. Experimental Details

Circular Dichroism Measurements and Melting Curves

CD spectra of the DNA strands were measured on a JASCO J-820 spectropolarimeter (Tokyo, Japan) in the Tris buffer (5 mM Tris-HCl, 0.5 mM EDTA, 100 mM NaCl, 20 mM KCl, 10 mM $MgCl_2$, pH 8.0) at room temperature. Spectra were recorded from 210 to 330 nm in 1 mm pathlength cuvettes and averaged from three scans. For CD melting experiments, 2 μM DNA strands in 10 mM lithium cacodylate buffer (100 mM NaCl, 20 mM KCl, 10 mM $MgCl_2$, pH 7.2) were heated from 20 °C to 90 °C at a rate of 1 °C/min in 10 mm pathlenth cuvettes. Thermal melting was monitored at 276 nm and 265.5 nm. UV melting curves of these DNA complexes at 260 nm were also collected on the J-820 spectropolarimeter at the same time.

Native polyacrylamide gel electrophoresis

DNA samples at the concentration of 10μM in Tris buffer with or without 20 mM K^+ were both heated at 88 °C for 5 min and then slowly cooled to room temperature before PAGE analysis. The DNA solutions mixed with 6 × loading buffer (10 mM Tris-HCl, pH 7.6, 50% glycerol, 0.25% bromphenol blue) were analyzed in 15% native polyacrylamide gel. The electrophoresis was conducted in 1 × TBE (89 mM Tris, 89 mM boric acid, 2 mM EDTA, pH 8.0) at 15 °C or in 1 × TBE with 20 mM K^+ at 4 °C. A constant voltage of 110V was used for 1 hour. Then the gel was stained with EB (ethidium bromide) for 20 min and briefly washed by water before visualization on a UV transilluminator.

S2. Study of the Background Signals

The fluorescence intensity caused by T30695 and the other long G-rich strands are compared in Fig. S1. Absence of the guanine bases in the strand indeed seriously influences the fluorescence enhancement ability of these G-rich strands, especially strand G8. The possible structures of long G-rich strands were subsequently analyzed by native polyacrylamide gel electrophoresis (PAGE) under different temperature and salt environment (Fig. S2). Three similar C-rich strands were added as control. Comparing the two gels and bands position of G-segments with control lane, we can easily find that the G-segments get less mobile at 4 °C with K^+, especially G6b. For the case of G6b, a dimeric G-quadruplex contained two G6b may be formed under this condition, which should be the reason of the highest background signal of split mode F 6:6 in Fig. 2. The bands of G9 and G10 also vary in different degree, which means some folded or self-assembled structures are formed. Changes of G7, G8 and G11 are not so apparent. For G11, its redundant guanine bases compared with other G segments may be an obstruction for its self-assembly. However, G11 owns 11 guanine bases and is only one guanine base less than T30695. It may still possess some basic factors to fold and interact with PPIX to some extent by itself. Thus the FI induced by G11 stays at a high level. For G7, the small difference between it and G8 may be not enough to lead visible change of bands in native PAGE. As shown in Fig. 3a, Fig. S1 and Fig. 2, the FI caused by G7 is the closest to that of G8 and the performance of split mode E is the second-best split mode after split mode D. Thus, split mode E is also a good choice and the native PAGE result of G7 is reasonable. Furthermore, to gain the exact structure of every conformer is unrealistic here.

What is certain from the fluorescence result is that the conformers of G8 induced the lowest fluorescence emission of PPIX in these structures.

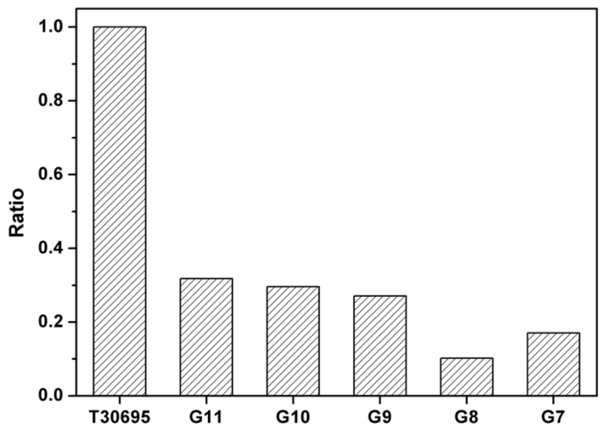

Fig. S1 Relative fluorescence intensity ratio of PPIX caused by T30695 and the other long G-rich strands at 630 nm.

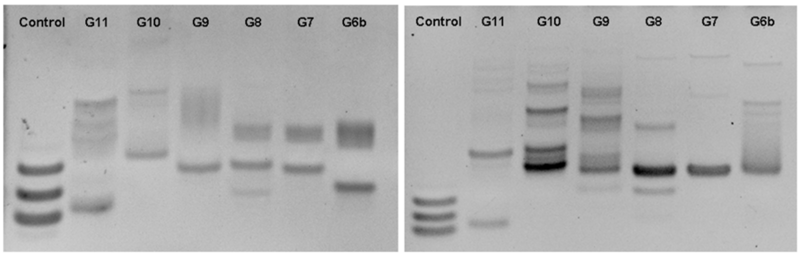

Fig. S2 Native polyacrylamide gel analysis (PAGE) of the long G-rich strands split from T30695. Electrophoresis was carried out at 15 °C without K^+ (left) and at 4 °C with 20 mM K^+ (right). Strand C6, C8 and C11 were mixed together and added in the first lane as control. The G-rich strands added in each lane have been indicated in the figure.

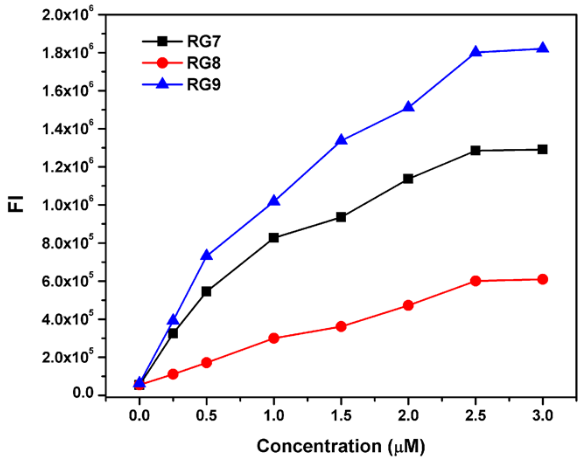

Fig. S3 Fluorescence titration of PPIX (1.0 μM) at 630 nm as a function of DNA G segments RG7, RG8 and RG9 concentrations.

S3. Application to Other G-quadruplexes

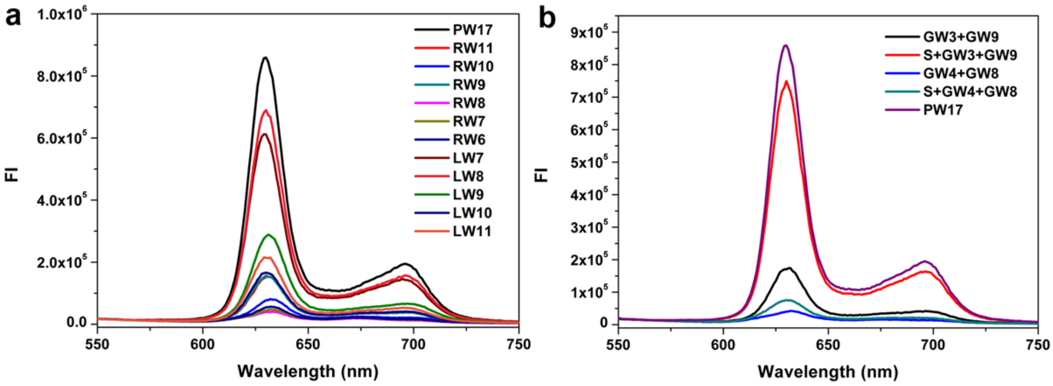

c

Sequence	PW17	RW11	RW10	RW9	RW8	RW7	RW6	LW7	LW8	LW9	LW10	LW11
Ratio	1	0.178	0.0900	0.183	0.0456	0.0522	0.0636	0.712	0.803	0.329	0.193	0.250

Fig. S4 (a) and (b) Fluorescence emission spectra of the complexes of PPIX and different DNA G-segments generated from splitting PW17. The strands used for each curve have been indicated in the plots. (c) Relative fluorescence intensity ratio of the FI of G segment to that of PW17 at 630 nm.

Another typical G-quadruplex sequence PW17 was also split and investigated by the same way. As shown in Fig. S4a and c, the signal of the long G segment RW8 gained by splitting PW17 from the 3’ end according to the 4:8 mode is the lowest among all the G-rich strands. This result demonstrates the magic “law of 4:8” was also practicable for PW17. However, the other bases among guanine bases in PW17 are not pure thymine like T30695, so not every split mode of PW17 can be easily recombined through the guidance of hybridization. In Fig. S4b, the G segments of PW17 generated by 4:8 split mode fails to enhance the fluorescence of PPIX with the guidance of the target strand, even though the 3:9 split mode works well here. This point should be taken into account when PW17 is used to design the split G-quadruplex based probe.

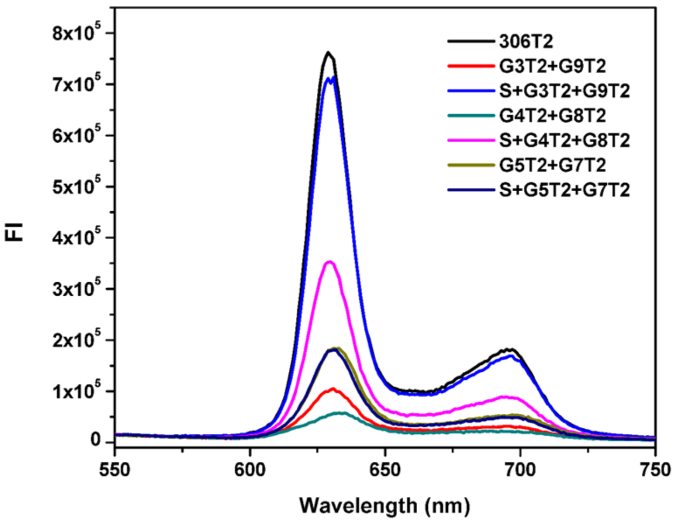

Fig. S5 Fluorescence emission spectra of the complexes of PPIX and different DNA G-segments generated from splitting 306T2. The strands used for each curve have been indicated in the figure.

We also inspected the conditions when the T loop of G-quadruplex got longer (GGGTTGGGTTGGGTTGGG). As shown in Fig. S5, the background signal generated from 4:8 split mode is still the lowest in the three modes, and ratio of positive signal to background is similar to that of mode 3:9. It is worth noting that G-segments split from 306T2 according to the 5:7 mode fails to enhance the fluorescence of PPIX when the target strand is present. The two examples above illustrate the background of the G segments generated by the split mode 4:8 is always the lowest and the bases in the loop domain will influence the ability of the recombined structure to enhance the fluorescence of PPIX.

S4. Effects of DNA Concentration and Salt Ions

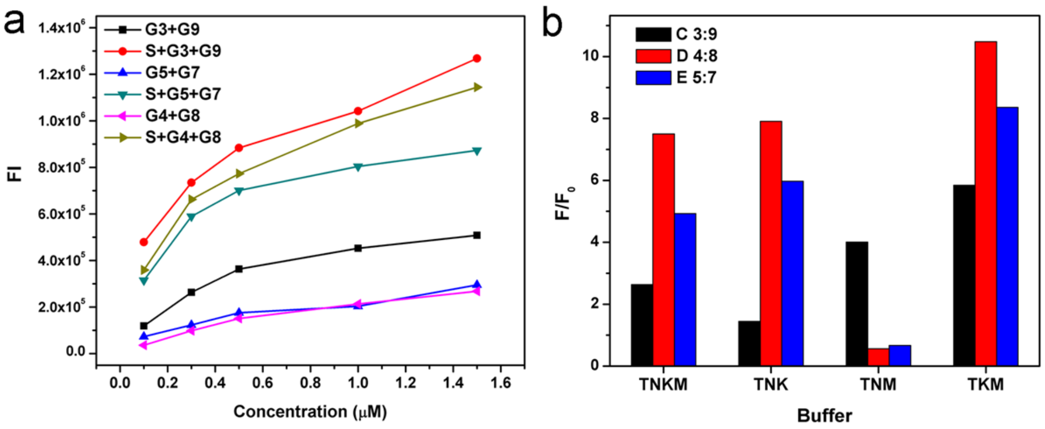

Fig. S6 (a) Fluorescence intensity of PPIX (0.5 μM) at 630 nm plotted against G segments and DNA complexes based on different split modes at various concentrations. (b) Ratio of signal to background for C, D and E split modes in four different buffers. Four capital letters T, N, K and M stand for 5 mM Tris, 100 mM NaCl, 20 mM KCl and 10 mM MgCl2 in the buffer (pH 8.0), respectively.

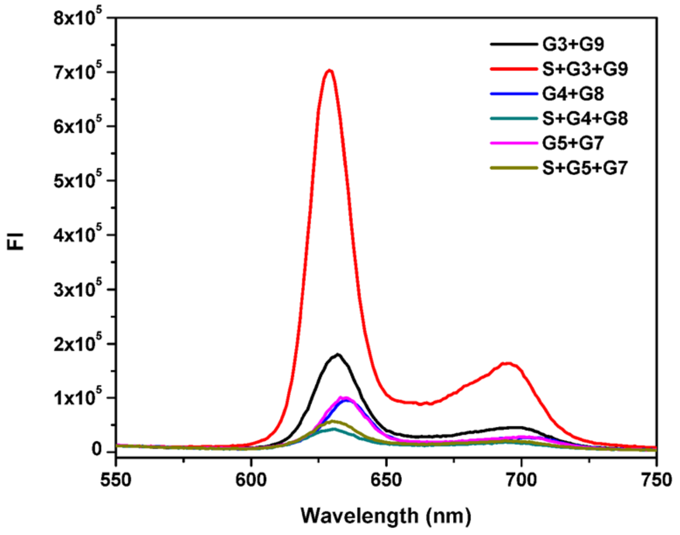

Fig. S7 Fluorescence emission spectra of the complexes of PPIX and DNA G-segments split by three different modes in TNM buffer. The strands used for each curve have been indicated in the plots.

Sodium and potassium ions are two important ions for the structure of G-quadruplex in solution, and magnesium ion play a key role in hybridization of DNA strands. Effects of these salt ions and DNA concentration for this sensing system are worth exploring. Mode C, D and E were still chosen as typical modes and inspected. The data in Fig. S6a demonstrate that the fluorescence of PPIX increases with the addition of G-segments and their target strand. The background signal of mode 4:8 keeps at a low level and its signal to background ratio is always the biggest for all concentrations. Unlike DNA concentration, the salt ions affect this split G-quadruplex enhanced fluorescence assay more. We investigated four different buffers in Fig. S6b. Through contrasting these ratios in different buffers, we found that the fluorescence result of split mode D and E is seriously influenced by potassium ion. When potassium ion is absent in TNM buffer, the fluorescence intensities of mode D and E are very low after the addition of target strand (Fig. S7). This phenomenon demonstrates that formation of the split G-quadruplex for G segments generated from split mode D and E relies heavily on the assistance of potassium ion, differing from mode C.

S5. Number of Guanine Bases in Split G-quadruplex

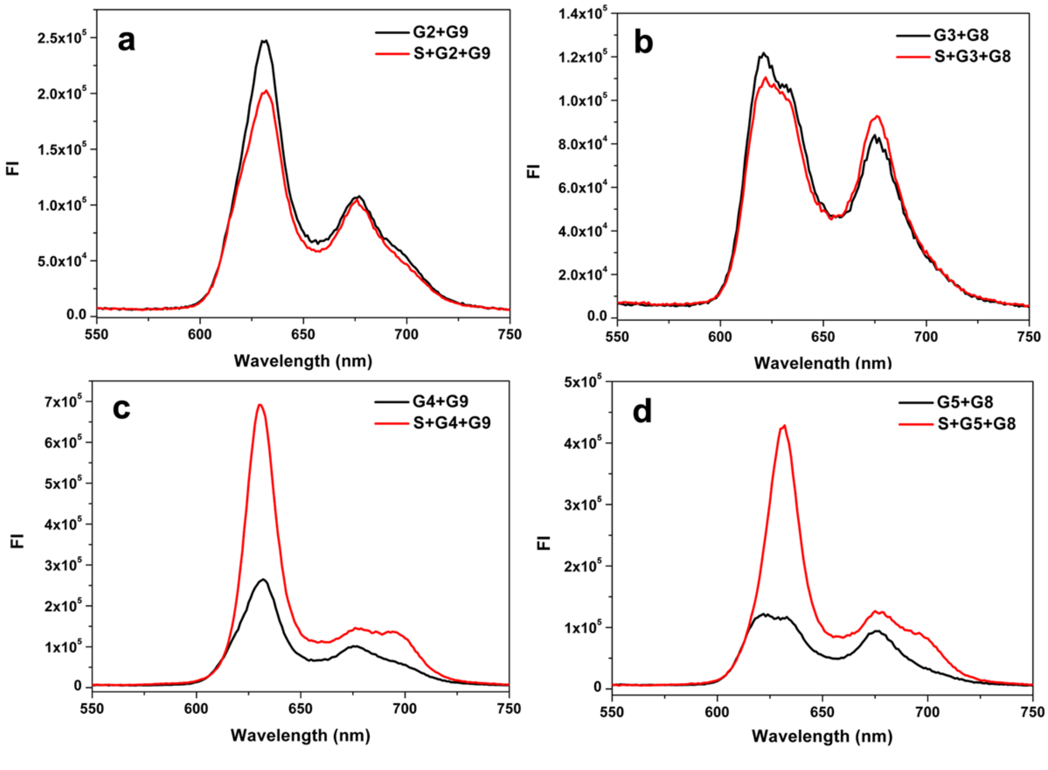

Fig. S8 Fluorescence emission spectra of the complexes of PPIX and different DNA G-segments. The strands used for each curve have been indicated in the plots.

From the above discussion we found that the number of guanine bases was very important for the formation of split G-quadruplex. In the case of mode A, absence of only one base of the four-stranded structure (G11) would dramatically affect the fluorescence result. In the reported works, the split G-quadruplex is usually made up by guanine bases

with a total number of twelve. What it will be like if more or less guanine bases are used to form the split G-quadruplex? To figure out the role of the guanine base number in the formation of the split G-quadruplex, we utilized different combinations of G-segments to rebuild G-quadruplex. The condition of eleven guanine bases was investigated at first. As shown in Fig. S8a and b, the fluorescence intensities of the two combinations (2:9 and 3:8) are both kept at a low level and even decrease after the addition of strand S. The decrease of the FI should be due to the hybridization between S and G-segments, which would inhibit the self-assembly of G9 or G8. The low FI identifies the importance of the integrity of the G-quadruplex structure in binding with its ligand. When the number of guanine base is abundant, the results are quite different. In Fig. S8c and d, the fluorescence emission of mode 4:9 and 5:8 are similar to the ones of mode C and D in Fig. 2, respectively. The difference is that the signals generated by the thirteen-guanine-base contained split G-quadruplex are a little weaker than twelve, especially mode 5:8 compared with mode D. This result demonstrated that split G-quadruplex can form when the guanine base number is more than twelve, but the extra guanine bases have some influence on the four-stranded structure and the enhanced fluorescence intensity.

S6. CD and UV Melting Studies

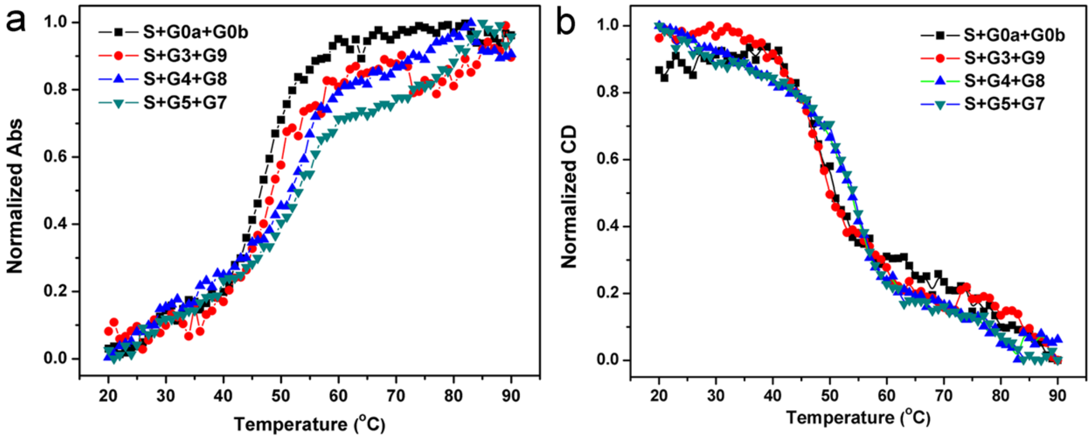

Fig. S9 (a) UV melting curves for four different DNA complexes (2.0 μM) at 260 nm. (b) CD melting curves for four different DNA complexes (2.0 μM) at 265.5 nm. The strands used for each curve have been indicated in the plots.

The UV (Fig. S9a) and CD melting curves (Fig. S9b) of these DNA complexes were also collected to study the change of G-quadruplex structure. Melting temperatures (T_m) evaluated from CD and UV melting curves were all given in Table S2. The data proved that the split G-quadruplex structure were dissociated with the denaturation of DNA duplex structure.

It is worth noting that DNA complex in mode C is less stable than mode D and E according to the results in Fig. 4 and Fig. S9. We think that it is due to the different effects of salt ions on them. As shown in Fig. S6b and Fig. S7, an obvious difference between split mode D/E and C is that the different effect of potassium ion on them. Formation of the split G-quadruplexes in split mode D and E relies heavily on the assistance of potassium ion, but the split

G-quadruplex in mode C can easily form in all buffers. Salt ions do not have much influence on the formation of split G-quadruplex in mode C. Their main function in this case is to assist the hybridization of DNA strands to form the three-strand complex. Thus, in the CD melting experiment, potassium ion must contribute much to the stability of the split G-quadruplexes in mode D and E as well as the whole DNA complex. However, the G segments based on split mode C seem to be freer than those in mode D and E. The split G-quadruplex in mode C can easily form in the absence of K^+, and so we believe that it can also easily disassemble even in the presence of K^+. Therefore, we think that the main reason for this phenomenon is the different effect of potassium ion on them.

S7. DNA Detection based on Split Mode C and E

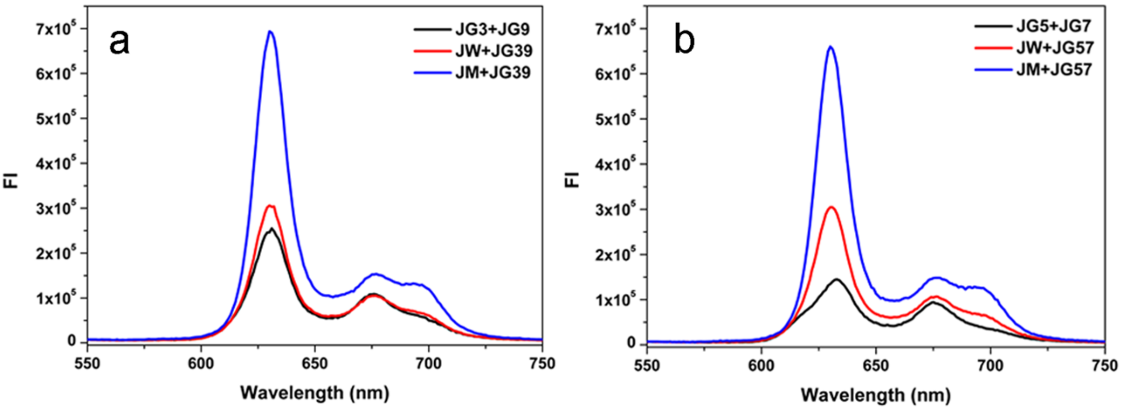

Fig. S10 Fluorescence emission spectra of the complexes of PPIX and DNA G-segments split by mode C (a) and mode E (b) for the detection of JAK2 V617F point mutation. The strands added for each curve have been indicated in the plots.

Fig. S11 Analysis of multiple target strands by a pool of G-segments. MixG represent a group of G-segments split by (a) mode C that consists of strands G3, G9, JG3, JG9, HG3 and HG9, and (b) mode E that consists of strands G5, G7, JG5, JG7, HG5 and HG7.

Tables

Table S1.* Sequences of the oligonucleotides used in this work.

Name	Sequence	Name	Sequence
S	GCAGCATCTC CTATTACGTC	RW7	GGGTAGGGCG
G0a	GAGATGCTGC	RW6	GGGTAGGGC
G0b	GACGTAATAG	LW7	GCGGGTTGGG
G1	GT GAGATGCTGC	LW8	GGCGGGTTGGG
G2	GGT GAGATGCTGC	LW9	GGGCGGGTTGGG
G3	TGGGT GAGATGCTGC	LW10	GTAGGGCGGGTTGGG
G4	GTGGGT GAGATGCTGC	LW11	GGTAGGGCGGGTTGGG
G5	GGTGGGT GAGATGCTGC	GW4	GTTGGGT GAGATGCTGC
G6a	GGGTGGGT GAGATGCTGC	GW8	GACGTAATAG TGGGTAGGGCGG
G6b	GACGTAATAG TGGGTGGG	GW3	TTGGGT GAGATGCTGC
G7	GACGTAATAG TGGGTGGGTG	GW9	GACGTAATAG TGGGTAGGGCGGG
G8	GACGTAATAG TGGGTGGGTGG	JW	AATTATGGAGTATG TGTCTGTGGAGACG
G9	GACGTAATAG TGGGTGGGTGGG	JM	AATTATGGAGTATG TTTCTGTGGAGACG
G10	GACGTAATAG TGGGTGGGTGGGTG	JG3	TGGGT CATACTCCATAA
G11	GACGTAATAG TGGGTGGGTGGGTGG	JG4	GTGGGT CATACTCCATAA
C6	GACGTAATAG TCCCTCCC	JG5	GGTGGGT CATACTCCATAA
C8	GACGTAATAG TCCCTCCCTCC	JG7	CTCCACAGAAA TGGGTGGGTG
C11	GACGTAATAG TCCCTCCCTCCCTCC	JG8	CTCCACAGAAA TGGGTGGGTGG
T30695	GGGTGGGTGGGTGGGT	JG9	CTCCACAGAAA TGGGTGGGTGGG
RG11	GGGTGGGTGGGTGG	Hbbw	GTGCACCTGACTC CTGAGGAGAAG
RG10	GGGTGGGTGGGTG	Hbbm	GTGCACCTGACTC CTGTGGAGAAG
RG9	GGGTGGGTGGGT	HG3	TGGGT GAGTCAGGTGCAC
RG8	GGGTGGGTGG	HG4	GTGGGT GAGTCAGGTGCAC
RG7	GGGTGGGTG	HG5	GGTGGGT GAGTCAGGTGCAC
RG6	GGGTGGGT	HG7	CTTCTCCACAG TGGGTGGGTG
LG7	GTGGGTGGGT	HG8	CTTCTCCACAG TGGGTGGGTGG
LG8	GGTGGGTGGGT	HG9	CTTCTCCACAG TGGGTGGGTGGG
LG10	GTGGGTGGGTGGGT	306T2	GGGTTGGGTTGGGTTGGG
LG11	GGTGGGTGGGTGGGT	G3T2	TGGGT GAGATGCTGC
PW17	GGGTAGGGCGGGTTGGG	G4T2	GTTGGGT GAGATGCTGC
RW11	GGGTAGGGCGGGTTGG	G5T2	GGTTGGGT GAGATGCTGC
RW10	GGGTAGGGCGGGTTG	G7T2	GACGTAATAG TGGGTTGGGTTG
RW9	GGGTAGGGCGGG	G8T2	GACGTAATAG TGGGTTGGGTTGG
RW8	GGGTAGGGCGG	G9T2	GACGTAATAG TGGGTTGGGTTGGGT

*All sequences start from 5' end. The bases in the blue color are designed to form the split G-quadruplex and the bases in the red color are the mutation sites.

Table S2. Table of T_m for four different DNA complexes evaluated from CD melting curves at 276 and 265.5 nm and UV melting curves at 260 nm.

T_m (°C)	S+G0a+G0b	S+G3+G9	S+G4+G8	S+G5+G7
CD at 276 nm	47	49	54	54
CD at 265.5 nm	48	49	54	55
UV at 260 nm	48	50	55	56

Table S3. Table of the FI ratios for different split modes in Figure 5 a, b and c.

Ratios	Mode C	Mode D	Mode E
Signal/Background	2.82	7.52	4.90
JM/JW	2.27	2.50	2.17

Table S4. Table of all abbreviations used in the manuscript and their explanation.

Abbreviation	Full term or interpretation
306T2	G-rich control strand with two thymine bases in the loop of T30695
Cx	C-rich control strand used in native PAGE. "x" represents the number of cytosine
CD	Circular dichroism
ESI	Electronic Supplementary Information
FI	Fluorescence intensity
Gx	G segments split from T30695 with a sensing arm for detection of strand S. "x" represents the number of guanine bases.
GxT2	G segments split from 306T2 with a sensing arm for detection of strand S. "x" represents the number of guanine bases.
GWx	G segments split from PW17 with a sensing arm for detection of strand S. "x" represents the number of guanine bases.
HBB	Hemoglobin, beta, also referred to β-globin, a globin protein
Hbbm	Mutation type target segment of HBB gene
Hbbw	Wild type target segment of HBB gene
HGx	G segments split from T30695 with a sensing arm for detection of Hbbm. "x" represents the number of guanine bases.
HGxy	A mixture solution of HGx and HGy for detection of Hbbm. "x" and "y" depend on the split mode.
JAK2	Janus kinase 2, a non-receptor tyrosine kinase
JM	Mutation type target segment of JAK2 gene
JW	Wild type target segment of JAK2 gene
JGx	G segments split from T30695 with a sensing arm for detection of JM. "x" represents the number of guanine bases.
JGxy	A mixture solution of JGx and JGy for detection of JM. "x" and "y" depend on the split mode.
LGx	Long G segments gained by splitting T30695 from the 5' end. "x" represents the number of guanine bases.

LWx	Long G segments gained by splitting PW17 from the 5' end. "x" represents the number of guanine bases.
mixG	A mixture solution of Gx, Gy, HGx, HGy, JGx and JGy for detection of S, Hbbm and JM at the same time. "x" and "y" depend on the split mode.
MPDs	Myeloproliferative disorders
MPIX	Mesoporphyrin IX, a porphyrin derivative
NMM	N-methyl mesoporphyrin IX, a porphyrin derivative
PAGE	Polyacrylamide gel electrophoresis
PPIX	Protoporphyrin IX, a porphyrin derivative
PW17	A common G-quadruplex
RGx	Long G segments gained by splitting T30695 from the 3' end. "x" represents the number of guanine bases.
RWx	Long G segments gained by splitting PW17 from the 3' end. "x" represents the number of guanine bases.
S+Gx+Gy	Three-strand complex formed upon the hybridization of sensing arms. "x" and "y" represent the number of guanine bases in corresponding G-rich strand.
SNP	Single nucleotide polymorphism
T30695	A common G-quadruplex studied in this work as a model.
WHO	World Health Organization
